# Effect of Sequential Acclimation to Various Carbon Sources on the Proteome of *Acetobacter senegalensis* LMG 23690^T^ and Its Tolerance to Downstream Process Stresses

**DOI:** 10.3389/fmicb.2019.00608

**Published:** 2019-03-26

**Authors:** Rasoul Shafiei, Pierre Leprince, Atena Sadat Sombolestani, Philippe Thonart, Frank Delvigne

**Affiliations:** ^1^Department of Biology, Faculty of Sciences, University of Isfahan, Isfahan, Iran; ^2^GIGA-Neurosciences, University of Liège, Liège, Belgium; ^3^Walloon Center for Industrial Biology, University of Liège, Liège, Belgium; ^4^Microbial Processes and Interactions, Gembloux Agro-Bio Tech, University of Liège, Gembloux, Belgium

**Keywords:** starter, acclimation, 2D-DiGE, *Acetobacter senegalensis* LMG 23690^T^, acetic acid, vinegar, stress, ethanol

## Abstract

Acetic acid bacteria are very vulnerable to environmental changes; hence, they should get acclimated to different kinds of stresses when they undergo downstream processing. In the present study, *Acetobacter senegalensis* LMG 23690^T^, a thermo-tolerant strain, was acclimated sequentially to different carbon sources including glucose (condition Glc), a mixture of glucose and ethanol (condition EtOH) and a mixture of glucose and acetic acid (condition GlcAA). Then, the effects of acclimation on the cell proteome profiles and some phenotypic characteristics such as growth in culture medium containing ethanol, and tolerance to freeze-drying process were evaluated. Based on the obtained results, despite the cells acclimated to Glc or EtOH conditions, 86% of acclimated cells to GlcAA condition were culturable and resumed growth with a short lag phase in a culture medium containing ethanol and acetic acid. Interestingly, if *A. senegalensis* LMG 23690^T^ had been acclimated to condition GlcAA, 92% of cells exhibited active cellular dehydrogenases, and 59% of cells were culturable after freeze-drying process. Proteome profiles comparison by 2D-DiGE and MS analysis, revealed distinct physiological status between cells exposed to different acclimation treatments, possibly explaining the resulting diversity in phenotypic characteristics. Results of proteome analysis by 2D-DiGE also showed similarities between the differentially expressed proteins of acclimated cells to EtOH condition and the proteome of acclimated cells to GlcAA condition. Most of the differentially regulated proteins are involved in metabolism, folding, sorting, and degradation processes. In conclusion, acclimation under appropriate sub-lethal conditions can be used as a method to improve cell phenotypic characteristics such as viability, growth under certain conditions, and tolerance to downstream processes.

## Introduction

Acetic acid bacteria (AAB) have been used in industrial production of vinegar, gluconic acid and L-sorbose (Singh and Kumar, 2007; De Roos and De Vuyst, 2018). They can potentially be used for the production of cellulose and other biological and pharmaceutical compounds (Adachi et al., 2003; Shafiei et al., 2017). Different strains of AAB are used in vinegar production. However, *Acetobacter* spp. and *Komagataeibacter* spp. are normally responsible for traditional surface vinegar production and high-acid vinegar production, respectively (Andrés-Barrao et al., 2016).

Thermophilic and thermo-tolerant AAB are valuable because they can reduce the cooling cost of bioreactors in industrial scale (Ndoye et al., 2006). In addition, they tolerate temperature fluctuations better than mesophilic AAB during fermentation (Ndoye et al., 2006). *Acetobacter senegalensis* LMG 23690^T^, a thermo-tolerant strain which is used through the present study, was first isolated from mango fruit. It is able to oxidize ethanol at 25–42°C (Ndoye et al., 2007a; Shafiei et al., 2013a). This strain was successfully used for production of vinegar and vinegar starter in pilot plant scale acetifier (Ndoye et al., 2007b; Shafiei et al., 2014). Interestingly, *Acetobacter senegalensis* LMG 23690^T^ produces acetic acid and gluconic acid simultaneously at high temperature (38°C) when grown on a mixture of ethanol and glucose (Mounir et al., 2016).

Different carbon sources can be used for the growth of AAB depending on the genera and species (Mamlouk and Gullo, 2013). *Acetobacter* spp. oxidize aerobically different carbon sources including ethanol, glycerol and glucose (Deppenmeier and Ehrenreich, 2009). Ethanol is oxidized to acetic acid. The produced acetic acid is further oxidized to CO_2_ and H_2_O (Sievers and Swings, 2005) via TCA cycle and glyoxylic acid shunt (Deppenmeier and Ehrenreich, 2009; Sakurai et al., 2011). Ethanol tolerance has been strongly correlated with adaptive changes in plasma membrane composition (Montooth et al., 2006; Trček et al., 2007). In addition, many SOS-response genes are up-regulated in *Acetobacter aceti* grown on ethanol, indicating that ethanol may provoke damage to DNA and proteins (Sakurai et al., 2011). Acetic acid as the main product of ethanol oxidation, is an uncoupling agent which damages or kills cells. Its detrimental impacts on cells are observed in lower pH, particularly below its ionization constant (p*K*a 4.74) (Nicolaou et al., 2010). Under particular conditions, over-oxidizer species of *Acetobacter* may utilize acetic acid as a carbon source (Mamlouk and Gullo, 2013). D-Glucose, as another carbon source, is also used by AAB especially *Gluconobacter* spp.; however, it is a less favorable carbon source for *Acetobacter* spp. (Mamlouk and Gullo, 2013).

Vinegar starter production have been extensively studied (Sokollek and Hammes, 1997; Sokollek et al., 1998; Ndoye et al., 2007b; Gullo and Giudici, 2008). The effects of different carbon sources on vinegar starter culture production were also investigated in some studies (Gullo et al., 2016). However, to the best of our knowledge, the influences of carbon sources used for biomass production, have not been studied on cell viability after freeze-drying process. Thus, the main aim of this study was to acclimate *Acetobacter senegalensis* LMG 23690^T^ to different carbon sources. Then, the influences of different growth conditions on the tolerance of cells to downstream processes was investigated. To understand how the acclimated cells resist against different kinds of stress during downstream process, their proteome profiles were compared by 2D-DiGE. Finally, the tolerance of acclimated biomass to freeze-drying process, and growth on ethanol were assessed.

## Materials and Methods

### Bacterial Strain and Culture Media

*Acetobacter senegalensis* CWBI-B418 (=LMG 23690^T^=DSM 18889^T^), a thermo-tolerant acetic acid bacterium was used throughout this study (Ndoye et al., 2006).

Three different culture media were used to culture *A. senegalensis* LMG 23690^T^.

(1) GY broth (glucose–yeast extract) was used to culture *A. senegalensis* LMG 23690^T^ on glucose. It contained: glucose 20 g/L, yeast extract 7.5 g/L, MgSO_4_.7 H_2_O 1 g/L, and (NH4)_2_HPO_4_ 1 g/L. Initial pH (3.9 ± 0.1) was adjusted by addition of 1.5N KOH.
(2) GYA broth (glucose–yeast extract–acetic acid) was used to culture *A. senegalensis* LMG 23690^T^ in the presence of glucose and acetic acid in low pH. Its components were identical to GY broth. In addition, 1–5% (w/v) acetic acid was added. Initial pH (3.9 ± 0.1) was adjusted by addition of KOH 9N.
(3) GYEA broth (glucose–yeast extract–ethanol–acetic acid) was used to culture *A. senegalensis* LMG 23690^T^ in the presence of ethanol, glucose, and acetic acid. It contained all the components of GY broth plus 5% (w/v) ethanol and 1% (w/v) acetic acid. Initial pH (3.9 ± 0.1) was adjusted by addition of KOH 9N.

To prepare solid culture media, 15 g/L agar was added to the above formulations. All culture media components were purchased from Merck^®^.

### Sequential Acclimation of *A. senegalensis* LMG 23690^T^ to Different Growth Conditions

**FIGURE 1:**
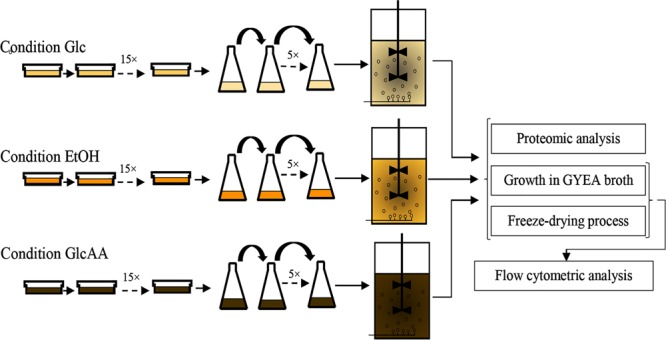
Experimental procedure used for the sequential acclimation of *Acetobacter senegalensis* LMG 23690^T^ to different carbon sources.

In order to acclimate *A. senegalensis* LMG 23690^T^ to different carbon sources, solid and broth culture media were used. The acclimation procedure is shown in Figure 1.

#### Acclimation on Solid Culture Media

To acclimate cells to glucose, acetic acid and initial low pH (3.9 ± 0.1), GYA agar supplemented with 1% (w/v) acetic acid was used. Cells were cultured on GYA agar. Then, several colonies were transferred to fresh GYA agar (with the same concentration of acetic acid) every 72 h. The concentration of acetic acid was progressively raised to 5% (w/v).

To acclimate cells to ethanol, acetic acid and initial low pH (3.9 ± 0.1), GYEA agar with 5% (w/v) ethanol and 1% (w/v) acetic acid was used. Cells were grown as described above and colonies were selected and transferred every 30–48 h.

To acclimate cells to glucose and low pH (3.9 ± 0.1), GY agar was used. Cells were cultured on GY agar. Several colonies were transferred to a fresh medium every 36 h.

For each culture medium, cells were passaged 15 times into fresh culture media (Figure 1). The plates were incubated at 30°C under aerobic condition.

#### Acclimation in Liquid Culture Media

Acclimated cells in GY agar and GYEA agar were inoculated to GY broth and GYEA broth, respectively. To acclimate cells to glucose, acetic acid and initial low pH, GYA broth containing 3% (w/v) acetic acid with initial pH 3.9 was used. Several colonies of GYA agar containing 3% acetic acid were transferred to it.

The flasks were then incubated at 30°C under aerobic condition (130 rpm, Laboshaker Grehardt^®^). When OD_540 nm_ reached value of 1.0 ± 0.05, 5 ml of the bacterial suspension was transferred to 95 ml of fresh medium. For each culture medium, cells were passaged five times into fresh culture media to get completely acclimated cells (Figure 1).

### Acclimation of *A. senegalensis* LMG 23690^T^ in Lab-Scale Bioreactor

#### Preparation of Pre-culture

Three different pre-cultures were prepared. Pre-culture was prepared in 5 L three-baffled flask containing 800 ml GYA broth or GY broth or GYEA broth. 20 ml of acclimated cell suspension (see section “Acclimation in Liquid Culture Media”) was used to inoculate the pre-culture. The flask was then incubated at 30°C under aerobic condition (120 rpm, Laboshaker Grehardt^®^) up to 36 h. When OD_540 nm_ reached value of 0.8 ± 0.05, they were used to inoculate the bioreactor.

#### Acclimation in Bioreactor

To grow acclimated *A. senegalensis* LMG 23690^T^ under regulated conditions, a Lab-scale bioreactor (Bio Lafitte France), with total volume of 15 L and working volume of 10 L was used. The bioreactor was equipped with three probes used for monitoring dissolved oxygen, temperature and pH. The aeration system consisted of two marine-blade impellers (left-handed orientation) and a cross-shaped sparger. The air inlet equipped with a flow meter was set at 1 vvm. The amount of dissolved oxygen was regulated by changing the marine-blade impeller rotation speed automatically according to the demanded oxygen during bacterial growth. The critical threshold for dissolved oxygen was set at 30%. Temperature was set at 30 ± 0.5°C.

Three different culture media (see section “Bacterial Strain and Culture Media”) were used to culture cells in bioreactor: (i) GY broth contained glucose as the main carbon source in regulated pH 3.9 ± 0.1 (this condition is referred as ‘Glc condition’ through this paper). (ii) GYA broth containing glucose and 3% (w/v) acetic acid as the main carbon sources in regulated pH 3.9 ± 0.1. The concentration of acetic acid was kept constant during fermentation (this condition is referred to as ‘GlcAA condition’ through this paper). (iii) GYEA broth containing ethanol and glucose as the main carbon sources in regulated pH 3.9 ± 0.1 (this condition is referred to as ‘EtOH condition’ through this paper).

The considered pH (3.9) was regulated by dosing pumps automatically using KOH (1.5 N) and H_3_PO_4_ (1.5 N) as regulator for condition ‘Glc condition’ and ‘EtOH condition,’ respectively. Regulation of pH for ‘GlcAA condition’ was done by acetic acid (10% w/v) and KOH (1.5N).

Each bioreactor was inoculated with its corresponding pre-culture medium (see section “Preparation of Pre-culture”). For each condition, three independent fermentation runs were performed.

### Determination of Glucose, Acetic Acid, and Ethanol Concentrations

Acetic acid, glucose, and ethanol concentrations during acclimation of cells in bioreactor were determined by HPLC as previously described (Shafiei et al., 2013a).

### Sample Preparation for Proteomic Analysis

**FIGURE 2:**
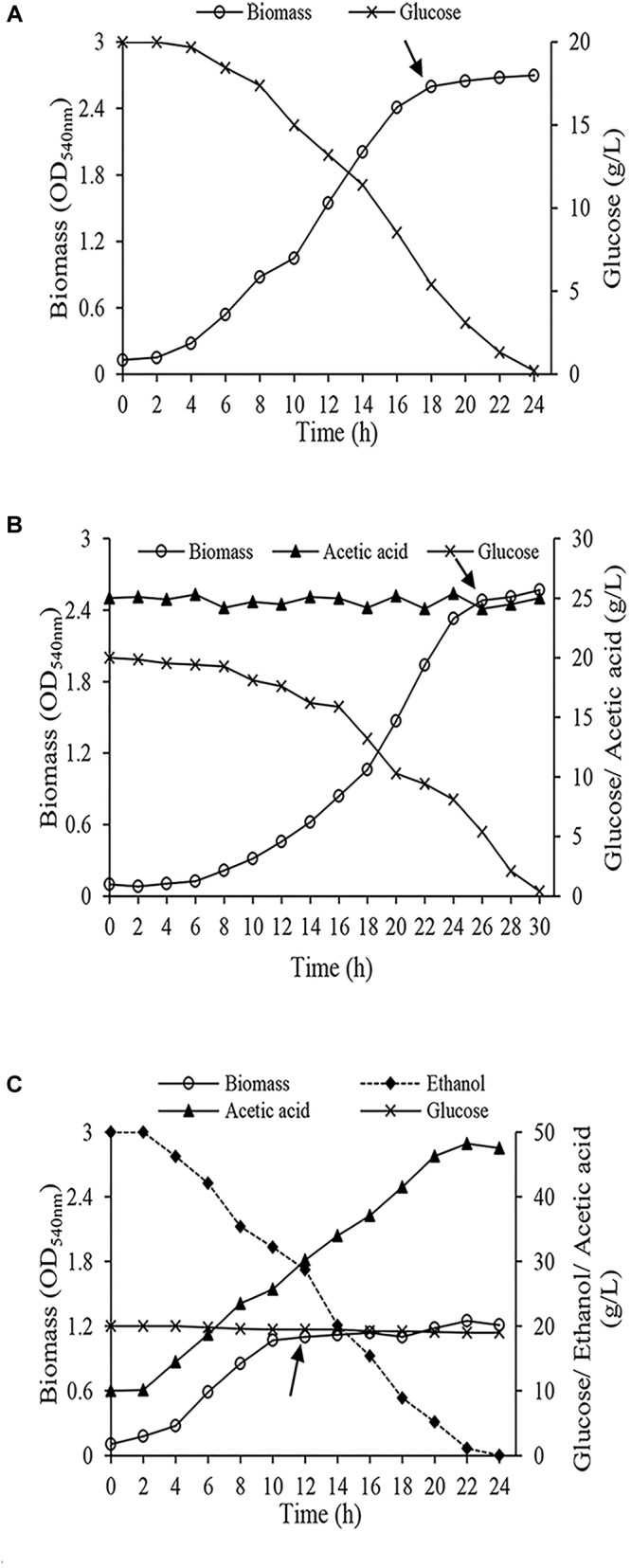
Growth of *Acetobacter senegalensis* LMG 23690^T^ in different carbon sources under regulated conditions (Lab-scale bioreactor, at 30°C and pH 3.9 ± 0.1) **(A)** cells acclimated to glucose (Glc condition), **(B)** cells acclimated to a mixture of ethanol and glucose (EtOH condition), **(C)** cells acclimated to a mixture of glucose and acetic acid (GlcAA condition). The arrows show the points where the cells were harvested for proteomic analysis and freeze-drying process. Results are representative of three independent experiments.

Cells were harvested from the early stationary phase (Figure 2) when the biomass reached the maximum amount. Harvested cells were centrifuged (4,000 × *g*, 10 min at 4°C) and washed twice with phosphate buffer solution (50 mM, pH 5.5). Finally, they were washed with distilled water. Washed cell were kept at -80°C.

### Two-Dimensional Difference Gel Electrophoresis (2D-DiGE)

Triplicate 2D-DiGE electrophoresis were carried out on acclimated cells according to the procedures previously described (Shafiei et al., 2014). Cells acclimated under the three different conditions (see section “Acclimation in Bioreactor”) were compared two by two. Protein spots which were different in abundance (±1.5-fold standardized abundance, *p* < 0.05) were picked and collected. They were then identified by mass spectrometry as described by Shafiei et al. (2014).

### Freeze-Drying of Produced Biomass and Storage

After production of biomass in bioreactors (see section “Acclimation in Bioreactor”), cells were harvested, washed with phosphate buffer solution (50 mM, pH 5.5) and concentrated (4,000 × *g*, 10 min at 4°C). The obtained creams were mixed with mannitol (20% w/w). It was then used for freeze-drying process under the conditions previously described by Shafiei et al. (2013b).

### Growth in Culture Media Containing Ethanol as the Main Carbon Source

To study the effect of acclimation on the subsequent growth in a culture medium containing ethanol, the harvested cells acclimated under the three above-described conditions (see section “Acclimation in Bioreactor”), were cultured in GYEA broth containing 5% ethanol (w/v) and 1% (w/v) acetic acid. The flasks were then incubated at 30°C under aerobic condition (130 rpm, Laboshaker Grehardt^®^). pH was not regulated during the growth of cells. Biomass production during different growth phases was determined by measuring OD_540 nm_. In addition acetic acid concentration was determined by HPLC method (see section “Determination of Glucose, Acetic Acid, and Ethanol Concentrations”).

### Assessment of Cell Culturability and Cell Viability

Spread plate technique was used to enumerate culturable cells. For this purpose, GYEA agar (see section “Bacterial Strain and Culture Media”) was used as the culture medium. Samples were diluted in GYEA broth and spread on GYEA agar followed by incubation at 30°C for 72–96. Total number of cells was determined by Bürker slide (Lo-laboroptic Ltd., Lancing, United Kingdom) using a phase contrast microscope (Olympus, Tokyo, Japan) (Shafiei et al., 2013a). Culturability is defined as the percentage of cells which grow on solid media under defined conditions (the ratio of culturable cells to the total number of cells).

Viability of freeze-dried cells was assessed by determination of total cellular dehydrogenase activity. Briefly, freeze dried cells were re-suspended in GYEA broth containing 5% (w/v) ethanol and 1% (w/v) acetic acid. Then, total cellular dehydrogenase activity was determined by flow cytometry, using 5-cyano-2,3-ditolyl tetrazolium chloride (CTC) as an indicator based on the procedure previously used by Shafiei et al. (2013a).

## Results and Discussion

### Acclimation of *A. senegalensis* LMG 23690^T^ to Different Carbon Sources

In the present study, based on an experimental design (Figure 1), *A. senegalensis* LMG 23690^T^ was acclimated sequentially to different culture media for achieving two practical purposes: (i) Improvement of cell survival during downstream process such as freeze-drying process. (ii) Enabling the acclimated and freeze-dried cells to grow rapidly on ethanol.

During acclimation on solid culture media, *A. senegalensis* LMG 23690^T^ grew differently. Fast growth was observed on GYEA agar. During the first five passages, colonies appeared after 42–48 h, and then colony formation time decreased to 30–36 h. Growth of *A. senegalensis* LMG 23690^T^ on GYA agar with different acetic acid concentrations showed that it grew in GYA agar containing maximum 3.5% (w/v) acetic acid in pH 3.9. Therefore, 3% (w/v) acetic acid was chosen to induce acetic acid stress. The time needed for the growth of colonies on GYA agar also decreased after several passages to 30–36 h.

In contrast to appropriate growth on GY agar and GYA agar, *A. senegalensis* LMG 23690^T^ exhibited the slowest growth on GY agar. Colony formation took about 72–80 h. In addition, colony formation time did not changed over sequential passages. This may be as a result of pH drop due to the oxidation of glucose to gluconic acid (Shafiei et al., 2017). Since pH and other fermentation parameters could not be monitored and regulated in solid culture media, acclimation in liquid culture media was used as a complementary method.

The growth features of *A. senegalensis* LMG 23690^T^ under regulated condition significantly was different from growth on solid and broth culture media. As it is seen in Figure 2C, cells grew rapidly under EtOH condition, and entered stationary phase while there was still about 3% (w/v) ethanol and about 3% (w/v) acetic acid in the medium.; however, the maximum amount of biomass was considerably lower than the two other conditions (Figure 2A,B). *A. senegalensis* LMG 23690^T^ consumed ethanol as the primary carbon source even in the presence of glucose (Figure 2C), therefore it can be inferred that bacterial physiology was considerably affected by ethanol and acetic acid. However, ethanol could not support the growth of *A. senegalensis* LMG 23690^T^ in the absence of glucose.

Growth under Glc condition and GlcAA condition was slower than EtOH condition; however, the amount of biomass produced under these two conditions was significantly higher (Figure 2A,B). Compared with Glc condition, *A. senegalensis* LMG 23690^T^ grown under condition GlcAA showed a longer exponential phase with lower growth rate. Since pH during fermentation was below the p*K*_a_ of acetic acid (4.76), most of the present acetic acid molecules were in undissociated form which enter the cells readily. Lower growth rate can be due to the metabolic load associated with ATP-dependent export mechanisms needed for expelling this molecule (Nakano et al., 2006; Segura et al., 2012).

To produce vinegar starter, it is necessary to obtain high amount of biomass. In addition, the biomass should be viable and vital. Therefore, we harvested cells in the beginning of stationary phase (Figure 2). They were then underwent freeze-drying process. In addition, a part of the harvested cells was used for proteomic analysis.

### Evaluation of Phenotypic Features of Acclimated *A. senegalensis* LMG 23690^T^ to Different Carbon Sources

#### Growth on Ethanol as the Main Carbon Source

Essentially, to produce vinegar starter, high amount of biomass is needed. In addition, to start vinegar production, vinegar starter is inoculated to a culture medium containing ethanol and acetic acid. Therefore, cells must be able to maintain viability, and grow quickly in a culture medium containing ethanol and acetic acid. As shown in Figure 2A,B, Glc condition and GlcAA supported the highest amount of biomass production based on the optical density measurement. In contrast, *A. senegalensis* LMG 23690^T^ did not produce abundant biomass in EtOH condition (Figure 2C). However, assessment of culturability of acclimated cells on GYEA agar containing 5% (w/v) ethanol and 1% (w/v) acetic acid exhibited different results. In agreement with the result of Figure 2C, the total number of cells which were produced under EtOH condition was six-times lower than the cells in Glc condition (Table 1). In addition, a large part of acclimated cells to EtOH condition were not culturable on GYEA agar. This result may indicate that ethanol acclimated cells are very susceptible to oxygen deprivation during harvesting process (Gullo et al., 2014). Moreover, although *A. senegalensis* LMG 23690^T^ exhibited the highest number of cells in Glc condition (Table 1), culturability of cells on GYEA agar was very low. In contrast, although GlcAA condition did not support optimum growth of cells (Figure 2B), the cultrability of cells was considerably higher (86%) than the two other conditions (Table 1). Sievers et al. (1992) reported the isolation of an industrial strain (*A. europaeus*) from high acid vinegar fermentations. In contrast to the other species of AAB, *A. europaeus* needs acetic acid for growth. In addition, it could produce higher amount of acetic acid. Thus, it can be deduced that growth in the presence of acetic acid may enhance the resistance of cells to subsequent stresses.

**Table 1:** Culturability of acclimated *Acetobacter senegalensis* LMG 23690^T^ to different conditions in culture medium containing ethanol and acetic acid at 30°C.

	Acclimation condition		
	GlcAA^a^	Glc^b^	EtOH^c^
Total number of cells (cells/ml)	7.45 × 10^8^ (±1.33 × 10^8^)	1.29 × 10^9^ (±1.91 × 10^8^)	2.1 × 10^8^ (±4.01 × 10^7^)
Culturable cells (CFU/ml)	6.42 × 10^8^ (±7.15 × 10^7^)	2.18 × 10^7^ (±3.0 × 10^6^)	1.9 × 10^1^ (±1.31)
Culturability^d^	86%	0%	0%

*^*a*^GlcAA condition: acclimation to a mixture of glucose and acetic acid in regulated pH (pH 3.9 ± 0.1), ^*b*^Glc condition: acclimation glucose in regulated pH (pH 3.9 ± 0.1), ^*c*^EtOH condition: acclimation to a mixture of glucose and ethanol in regulated pH (pH 3.9 ± 0.1). ^*d*^Culturability: the ratio of culturable cells to the total number of cells. Results are representative of three independent experiments and are expressed as mean ± SD (error bars) of three replicates.*

**FIGURE 3:**
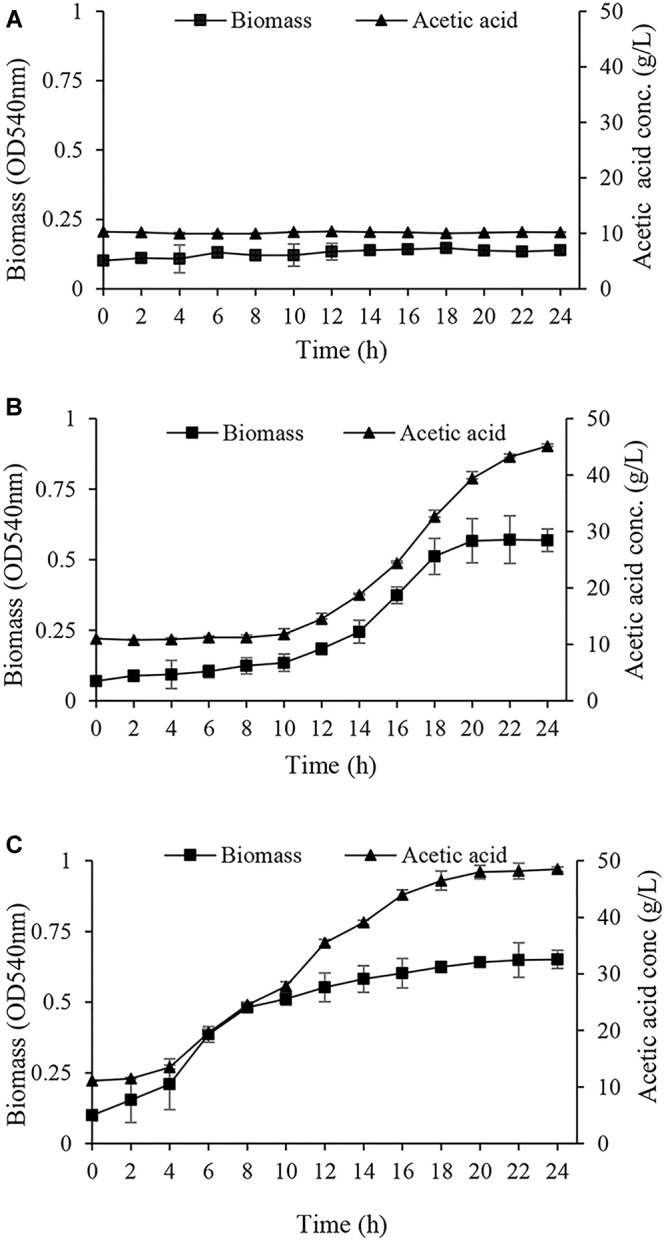
Growth and acetic acid production of *Acetobacter senegalensis* LMG 23690^T^ acclimated to different carbon sources. Acclimated cells were harvested and washed, then they were inoculated into GYEA broth containing 5% (w/v) ethanol and 1% (w/v) acetic acid. Results are representative of three independent experiments and are expressed as mean ± SD (error bars) of three replicates. **(A)** Cells acclimated to EtOH condition did not grow during 24 h. **(B)** Cells acclimated to glucose (Glc condition) started to grow after a long (10 h) lag phase. **(C)** Cells acclimated to a mixture of glucose and acetic acid (GlcAA condition) resumed the growth after a very short lag phase.

In the next step, the acclimated cells were harvested and inoculated into GYEA broth containing 5% (w/v) ethanol and 1% (w/v) acetic acid, and then the growth was monitored. Acclimated cells to EtOH condition were not able neither to grow nor to produce acetic acid during 24 h (Figure 3A). This inability to grow may arise from susceptibility to harvesting process and oxygen deprivation which can cause ethanol acclimated cells lose viability (Gullo et al., 2014). Cells acclimated to glucose (Glc condition) started to grow after a long lag phase (Figure 3B). In agreement with the results of Table 1, it can be deduced that the long lag phase may be due to the existence of a large number of un-acclimated sub-population which neither consumed ethanol nor tolerated acetic acid. Interestingly, *A. senegalensis* LMG 23690^T^ acclimated to GlcAA condition started to grow in GYEA broth with a short lag phase, and produce 5% acetic acid in 24 h (Figure 3C).

#### Tolerance to Freeze-Drying Process

**FIGURE 4:**
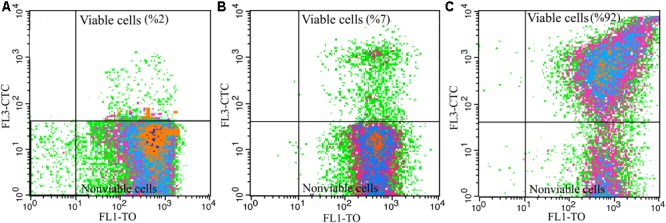
Influence of freeze-drying process on viability of *A. senegalensis* LMG 23690^T^ acclimated to different carbon sources. **(A–C)** Show viability of freeze-dried cells which were already acclimated to EtOH, Glc and GLCAA conditions, respectively. Cells were acclimated to different carbon sources. They were then harvested and underwent a freeze-drying process. Total dehydrogenase activity of freeze-dried cells were determined in a culture medium containing 5% (w/v) ethanol and 1% (w/v) acetic acid using CTC as an indicator.

To produce dried vinegar starter, acclimated cells to different carbon sources were underwent freeze-drying process. As it is shown in Figure 4A, and in agreement with the results of Table 1 and Figure 3A, the main part of cells (98%) acclimated to EtOH condition was not capable of reducing CTC after freeze-drying process. In other words, cells acclimated to EtOH, could not tolerate downstream process and lost dehydrogenase activity.

In line with the previous study (Shafiei et al., 2013b), it was observed that most of the cells (93%) acclimated to Glc condition were not viable after freeze-drying process (Figure 4B).

Notably, acclimation to GlcAA condition improved the tolerance of cells to freeze-drying process. As it is shown in Figure 4C, almost all the freeze-dried cells (92%) showed active dehydrogenases after freeze-drying process. In addition, about 59% of the cells were able to form colonies on GYEA agar containing 5% (w/v) ethanol and 1% (w/v) acetic acid. Previous studies have repeatedly demonstrated that pre-exposure to sub-lethal stress such as acid stress or high concentration of NaCl leads to improve the survival rate and activity of freeze-dried lactic acid bacteria (Barbosa et al., 2015; Li et al., 2015). However, to the best of our knowledge, this is the first study which shows acclimation to acetic acid increases the tolerance to freeze-drying process.

### Comparative Proteomic Analysis of Cells Acclimated to Different Carbon Sources

**FIGURE 5:**
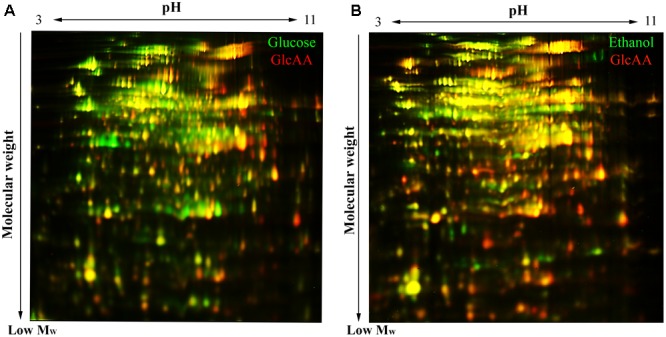
2D-DiGE gel images illustrating the difference of proteome composition of *A. senegalensis* LMG 23690^T^ acclimated to different carbon sources. **(A)** Comparison of cells acclimated to glucose (green spots) and cells acclimated to a mixture of glucose and acetic acid (red spots). **(B)** Comparison between the cells acclimated to EtOH condition (green spots) and the cells acclimated to a mixture of glucose and acetic acid (red spots). The yellow spots indicate the overlapping proteins (red and green spots) with similar abundance in both conditions.

Analysis of the 2D-DIGE gel sets by the DeCyder program (version 7.0; GE Healthcare) showed that 450 protein spot were consistently present in gel images of Glc, GlcAA and EtOH conditions (Figure 5). Considering a ±1.5-fold change of abundance and a *T*-test (*p* < 0.05) to be the threshold for inclusion, 107 protein spots had significant normalized abundance differences between samples acclimated to different carbon sources. All these protein spots are listed in the Supplementary Table S1.

Comparison between EtOH condition and Glc condition showed that 86 protein spots were differentially expressed. Among them, 72 proteins were up-regulated under EtOH condition and 14 proteins were up-regulated under Glc condition (Supplementary Table S1). The proteome comparison of cells acclimated to Glc condition with the cells acclimated to GlcAA condition showed that 67 protein spots were differentially expressed, and among them, 21 were up-regulated under Glc condition whereas 46 were up-regulated under GlcAA (Supplementary Table S1). Comparison between EtOH condition and GlcAA condition showed that 79 protein spots were expressed differentially, 59 of them were up-regulated under EtOH and 20 proteins were up-regulated under GlcAA (Supplementary Table S1).

As seen in Table 2, proteins involved in several metabolic pathways were the most frequent regulated proteins in all conditions. Moreover, the presence of ethanol or acetic acid in the culture media caused frequent up-regulation in proteins responsible for protein folding, sorting and degradations process. Interestingly, examination of Supplementary Table S1 reveals that about 42% of up-regulated proteins in GlcAA-Glc comparison are also up-regulated in the EtOH-Glc comparison. In other words, growth of *A. senegalensis* LMG 23690^T^ in the presence of ethanol (condition EtOH) or a mixture of acetic acid and glucose (condition GlcAA), induced up-regulation of similar proteins. Importantly, based on Table 2, it is revealed that cells acclimated to ethanol (condition EtOH) or a mixture of glucose and acetic acid (condition GlcAA), exhibited comparable up-regulation and down-regulation of more than 70% of metabolic proteins and more than 50% of proteins involved in folding, sorting, degradation process.

**Table 2:** Functional categories and fold change of differentially regulated proteins identified in *Acetobacter senegalensis* LMG 23690^T^ acclimated to different carbon sources.

Number	Functional categories	Proteins	Fold change
***A. senegalensis* LMG 23690^T^ acclimated to EtOH condition relative to *A. senegalensis* LMG 23690^T^ acclimated to Glc condition (EtOH>Glc).**			
1	Metabolism	Arylesterase	1.6–73.93
2	Metabolism	Aldehyde dehydrogenase	3.3–11.6
3	Metabolism	Alcohol dehydrogenase NADH-dependent iron-containing dependent	3.75
4	Metabolism	Acetyl-CoA hydrolase	2.06
5	Metabolism	Acetolactate synthase large subunit	3.6
6	Metabolism	Aldehyde/betaine dehydrogenase	3.37
7	Metabolism	Aldo/keto reductase	2.1–2.31
8	Metabolism	Acetolactate synthase large subunit	3.6
9	Metabolism	Acetyl-CoA decarbonylase/synthase gamma subunit	2.6
10	Metabolism	Fumarate hydratase	5.85–6.04
11	Metabolism	Dihydrolipoamide dehydrogenase	2.31
12	Metabolism	Inosine-5′-monophosphate dehydrogenase	2.63
13	Metabolism	NADH:flavin oxidoreductase	3.87
14	Metabolism	NAD(P) transhydrogenase subunit alpha	5.34
15	Metabolism	2-Oxoglutarate dehydrogenase E2 component	2.62–2.99
16	Metabolism	Ppx/GppA phosphatase	2.17
17	Metabolism	Succinyl-CoA:acetate coenzyme A transferase (SCACT)	1.78
18	Metabolism	Superoxide dismutase (SOD)	1.62
19	Metabolism	Succinate dehydrogenase Fe–S protein subunit	3.58
20	Metabolism	Glyceraldehyde 3-phosphate dehydrogenase	1.71
21	Folding, sorting, and degradation	Heat shock protein Hsp20/HspA	29.9
22	Folding, sorting, and degradation	Heat shock protein HtpG/Hsp90	4.41–5.99
23	Folding, sorting, and degradation	Molecular chaperone DnaK/Hsp70	3.41–5.61
24	Folding, sorting, and degradation	Clp protease ATP-binding subunit ClpB	2.96–4.66
25	Folding, sorting, and degradation	Chaperonin GroEL	2.25–2.57
26	Folding, sorting, and degradation	Peptidyl-prolyl *cis–trans* isomerase trigger factor	3.75
27	Folding, sorting, and degradation	Cold shock proteins	2.03
***A. senegalensis* LMG 23690^T^ acclimated to EtOH condition relative to *A. senegalensis* LMG 23690^T^ acclimated to Glc condition (EtOH<Glc).**			
28	Metabolism	Isocitrate dehydrogenase	1.53
29	Metabolism	Pyruvate phosphate dikinase	2.55
30	Metabolism	*S*-Adenosylmethionine (SAM) synthetase	3.85
31	Metabolism	Serine hydroxymethyl transferase	1.6
32	Folding, sorting, and degradation	Glutaredoxin	3.79
***A. senegalensis* LMG 23690^T^ acclimated to GlcAA condition relative to *A. senegalensis* LMG 23690^T^ acclimated to Glc condition (GlcAA<Glc)**			
33	Metabolism	Glutamine synthetase	1.61
34	Metabolism	Phosphopyruvate hydratase	2.08–2.58
35	Metabolism	Pyruvate phosphate dikinase	1.62–2.29 2.19
36	Metabolism	*S*-Adenosylmethionine (SAM) synthetase	2.81
37	Metabolism	Serine hydroxymethyl transferase	1.75
38	Folding, sorting, and degradation	Glutaredoxin	2.23
39	Folding, sorting, and degradation	Thioredoxin	5.38
***A. senegalensis* LMG 23690^T^ acclimated to GlcAA condition relative to *A. senegalensis* LMG 23690^T^ acclimated to Glc condition (GlcAA>Glc)**			
40	Metabolism	Acetyl-CoA hydrolase	3.17
41	Metabolism	Aldo/keto reductase	3.97–7.39
42	Metabolism	Acetyl-CoA decarbonylase/synthase gamma subunit	2.27
43	Metabolism	Aldehyde/betaine dehydrogenase	3.47
44	Metabolism	Acetolactate synthase large subunit	5.27
45	Metabolism	Dihydrolipoamide dehydrogenase	6.36
46	Metabolism	Fumarate hydratase	4.81–10.07
47	Metabolism	Isocitrate dehydrogenase	5.94
48	Metabolism	Inosine-5′-monophosphate dehydrogenase	2.54
49	Metabolism	NADH:flavin oxidoreductase	4.2
50	Metabolism	NAD(P) transhydrogenase subunit alpha	1.95
51	Metabolism	Osmotically inducible protein OsmC	1.77
52	Metabolism	2-Oxoglutarate dehydrogenase E2 component	4.77–4.83
53	Metabolism	Ppx/GppA phosphatase	3.63
54	Metabolism	Succinyl-CoA:acetate coenzyme A transferase (SCACT)	4.77
55	Metabolism	Succinate dehydrogenase Fe–S protein subunit	3.09–3.43
56	Metabolism	Superoxide dismutase (SOD)	3.32
57	Folding, sorting, and degradation	Heat shock protein Hsp20/alpha/HspA	3.56
58	Folding, sorting, and degradation	Heat shock protein HtpG/Hsp90	1.91
59	Folding, sorting, and degradation	Cold shock proteins	1.76
60	Folding, sorting, and degradation	DNA recombinase RecA	1.80
61	Folding, sorting, and degradation	Molecular chaperone DnaK/Hsp70	1.59–2.27
62	Folding, sorting, and degradation	Thioredoxin	2.27
63	Folding, sorting, and degradation	Peptidyl-prolyl *cis–trans* isomerase trigger factor	2.47
***A. senegalensis* LMG 23690^T^ acclimated to EtOH condition relative to *A. senegalensis* LMG 23690^T^ acclimated to GlcAA condition (EtOH>GlcAA).**			
64	Metabolism	Arylesterase	2.08–32
65	Metabolism	Alcohol dehydrogenase NADH-dependent iron-containing	5.42–6.2
66	Metabolism	Aldehyde dehydrogenase	3.32–5.51
67	Metabolism	Phosphopyruvate hydratase (enolase)	2.11–2.68
68	Metabolism	Pyruvate phosphate dikinase	2.11
69	Metabolism	Fructose-bisphosphate aldolase	1.55
70	Folding, sorting, and degradation	Heat shock protein Hsp20/alpha/HspA	1.84–8.4
71	Folding, sorting, and degradation	Heat shock protein HtpG/Hsp90	2.31–3.55
72	Folding, sorting, and degradation	Heat shock protein GroEL	2.33–2.49
73	Folding, sorting, and degradation	Clp protease ATP-binding subunit ClpB	2.59–3.54
74	Folding, sorting, and degradation	Glutaredoxin	2–5.24
75	Folding, sorting, and degradation	Molecular chaperone DnaK/Hsp70	1.52–2.89
76	Folding, sorting, and degradation	thioredoxin	4.38
***A. senegalensis* LMG 23690^T^ acclimated to GlcAA condition relative to *A. senegalensis* LMG 23690^T^ acclimated to EtOH condition (GlcAA>EtOH).**			
77	Metabolism	Isocitrate dehydrogenase	3-3.29
78	Metabolism	Glyceraldehyde 3-phosphate dehydrogenase	1.85-2.54
79	Metabolism	Osmotically inducible protein (OsmC)	2.04
80	Metabolism	Succinate dehydrogenase Fe–S protein subunit	2.03-3.29
81	Metabolism	2-Oxoglutarate dehydrogenase E2 component	1.66
82	Metabolism	Outer membrane protein (OmpA)	2.9
83	Metabolism	Succinyl-CoA:acetate coenzyme A transferase	2.68
84	Folding, sorting, and degradation	Thioredoxin	1.95

### Proteins Involved in Folding, Sorting, and Degradation Processes

In the present study, we found that a group of proteins expressed differentially belonged to the proteins involved in folding, sorting and degradation processes (Table 1). The up-regulated proteins were the members of heat shock protein family including: DnaK, GroEL (Hsp60), HtpG (Hsp90), GrpE (Hsp20/Alpha/HspA). Besides, ClpB (htpM) which is a product of the degradation of the Clp protein, was also up-regulated mainly in the presence of ethanol (EtOH condition) (Table 2).

It has been previously reported that ethanol stress increases the expression of DnaK and GroEL in *Bacillus subtilis* (Seydlova et al., 2012), *Lactobacillus sakei* (Schmidt et al., 1999). Our results also showed significant increases of DnaK, GroEL, Hsp90 and GrpE in the presence of ethanol (Table 2). In contrast, the presence of acetic acid in culture medium just induced DnaK, Hsp20, and Hsp90; but not GrpE and GroEL. These results are consistent with the results obtained by Okamoto-Kainuma et al. (2004) who demonstrated that mRNA levels of DnaK/J increases in *Acetobacter aceti* after the exposure to ethanol or acetic acid. However, they concluded that over expression of DnaK/J did not increase the acetic acid tolerance (Okamoto-Kainuma et al., 2004). Additionally, we observed that the cells acclimated to EtOH condition showed over-expression of GroEL whereas the cells acclimated to GlcAA did not show significant increase in GroEL. Still the latter were resistant to acetic acid and also could resume rapid growth in the presence of ethanol. This indicates that although ethanol could induce GroEL over expression, it might not be necessary for tolerance to ethanol. Andrés-Barrao et al. (2012) also concluded that growth of *Acetobacter pasteurianus* on ethanol in the presence of high acetic acid, induces overexpression of GroELS considerably which is consistent with our results.

Moreover, Okamoto-Kainuma et al. (2004) showed that *A. aceti* with GroEL and GroES overexpression, was more resistance to stresses such as ethanol, acetic acid and high temperature than wild *A. aceti* (Akiko et al., 2002). Thus, it can be deduced that, although over expression of GroEL/S can increase the resistance of AAB to acetic acid, wild type of AAB express these genes when ethanol is oxidized to acetic acid and accumulated in culture medium.

As already mentioned and shown in Table 2, GrpE did not show up-regulation in the presence of acetic acid (GlcAA condition), whereas an increase in GrpE was observed when ethanol was used as main substrate (EtOH). It was already shown that overexpression of GrpE or co-overexpression of GrpE with DnaK/J in *Acetobacter pasteurianus* resulted in improved growth compared to the single overexpression of DnaK/J in high temperature or ethanol-containing conditions, but no change in the growth profile of overexpressed strain was observed in the presence of acetic acid (Ishikawa et al., 2010). In agreement with this result, it can be inferred that GrpE acts for resistance to stress during ethanol oxidation by AAB (Ishikawa et al., 2010).

We found that ClpB was up-regulated in the presence of ethanol (Table 2). This protein is the main essential heat-shock response protein, which rescues stress-damaged proteins from an aggregated state (Lee et al., 2003). It has been already shown that ClpB expression is induced after exposure of bacteria to heat stress (Pan et al., 2012), ethanol and low pH (Ekaza et al., 2001). Ishikawa et al. (2010) found that ClpB is overexpressed in *A. pasteurianus* grown on ethanol or at high temperature. Still, by using 3% (w/v) acetic acid, they could not detect ClpB expression (Ishikawa et al., 2010). Andrés-Barrao et al. (2012) did not report the up-regulation of ClpB in cells grown in glucose and transferred to ethanol. This may be related to some limitations of this technique or difference between studied species (Andrés-Barrao et al., 2012).

We also found a slight increase in peptidyl-prolyl *cis–trans* isomerase (PPIase) in the presence of ethanol (Table 2). PPIase is a trigger factor (TF) (Hoffmann and Bukau, 2009) which may be involved in some bacterial functions such as virulence (Reffuveille et al., 2012), tolerance to acid and oxidative stress (Wen et al., 2005). Andrés-Barrao et al. (2012) did not detect this protein during oxidative fermentation of ethanol, a condition which was similar to the EtOH condition in our experiments.

RecA protein exhibited an over expression when *A. senegalensis* LMG 23690^T^ was acclimated to a mixture of glucose and acetic acid (Table 2). RecA is involved in the SOS response to DNA damage. The up-regulation of this protein in combination with the other mentioned stress-response proteins, indicates that the cells acclimated to ethanol or acetic acid may suffer from damage to proteins or DNA. This may be due to the toxicity of acetaldehyde produced during oxidation of ethanol that is known to induce DNA damage and protein denaturation (Sakurai et al., 2011).

### Proteins Involved in Metabolism

*Acetobacter senegalensis* LMG 23690^T^ was able to produce biomass using the three different carbon sources. This finding is in accordance with previous studies documented the growth of *Acetobacter* spp. on these sources (Gullo and Giudici, 2008; Andrés-Barrao et al., 2012; Gullo et al., 2012). In addition, although EtOH condition supported the growth of *A. senegalensis* LMG 23690^T^, it considerably inhibited the biomass formation which is attributed to the inhibitory effect of acetic acid following ethanol oxidation.

According to previous studies on some typical strains of AAB, Embden-Meyerhof-Parnas (EMP) pathway, TCA cycle, pentose-phosphate pathway (PPP) and glyoxylate pathway, exist in *Acetobacter* spp. such as *A. aceti* and *A. pasteurianus* (Saeki et al., 1999; Mullins et al., 2008; Sakurai et al., 2011). Additionally, most of PPP and TCA cycle genes are constitutively expressed or showed higher expression levels in the culture of *Acetobacter aceti* on acetate or glucose (Sakurai et al., 2011, 2012). Moreover, most of the EMP pathway genes, are constitutively expressed when *Acetobacter aceti* was grown on ethanol. The genes for glyoxylate pathway enzymes were significantly up-regulated soon after the cells began oxidizing ethanol indicating the importance of this pathway for utilization of ethanol as a carbon source (Sakurai et al., 2012). In the present study, as shown in Table 2, some of the EMP pathway proteins such as glyceraldehyde 3-phosphate dehydrogenase (GAPDH) and fructose-1,6-bisphosphate aldolase, were up-regulated in the presence of a mixture of ethanol and glucose (comparison EtOH-Glc) and acetic acid (comparison EtOH-GlcAA), respectively (Table 2). In addition, some of the TCA cycle proteins such as fumarase, succinate dehydrogenase, oxoglutarate dehydrogenase, isocitrate dehydrogenase, and exhibited significant up-regulation when the cells acclimated to EtOH condition or GlcAA condition (Table 2). Thus, it can be deduced that the above mentioned biochemical pathways are running in *Acetobacter senegalensis* LMG 23690^T^ under the experimental conditions of this study.

**FIGURE 6:**
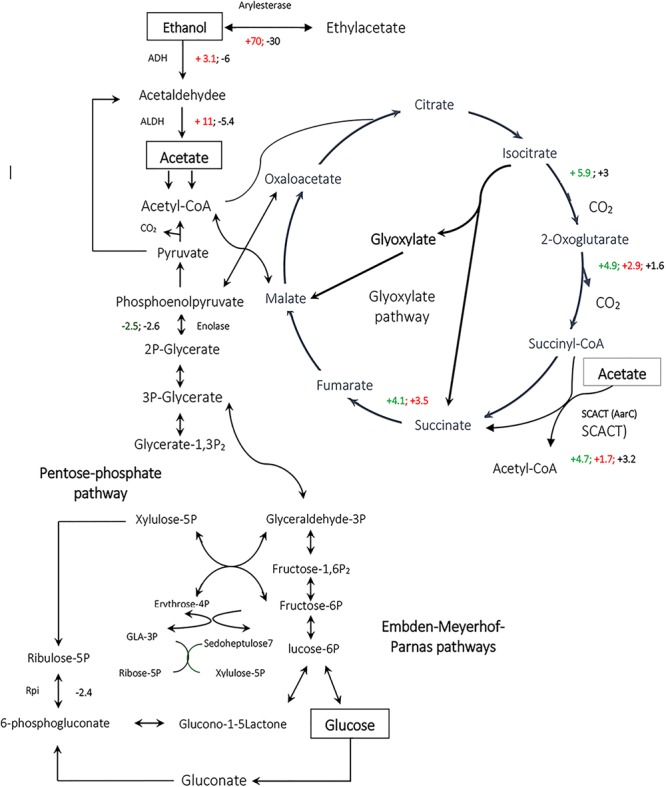
Comparative analysis of the expression of enzymes involved in metabolic pathways for *Acetobacter* spp. *Acetobacter senegalensis* LMG 23690^T^ was able to consume a mixture of glucose and ethanol (condition EtOH), glucose (condition Glc) or a mixture of glucose and acetic acid (condition GlcAA) at 30°C and pH 3.9. By comparison of each condition with two other conditions, we could determine the significant up-regulated or down-regulated proteins. The up-regulated proteins in ethanol condition have been shown as red positive numbers whereas the up-regulated proteins in Glc conditions have been shown as red negative numbers. GlcAA-Glc represents the comparison between GlcAA and Glc conditions. The up-regulated proteins in GlcAA condition have been shown as green positive numbers whereas the up-regulated proteins in Glc condition have been shown as green negative numbers. GlcAA-EtOH represents the comparison between GlcAA and EtOH conditions. The up-regulated proteins in Glc AA condition have been shown as black positive numbers whereas the up-regulated proteins in EtOH condition have been shown in black negative numbers.

Interestingly, in the present study, no change in abundance of succinyl-CoA synthetase (EC: 6.2.1.4 and 6.2.1.5) was detected in TCA cycle. Instead, succinyl-CoA:acetate coenzyme A transferase (SCACT) (acetic acid resistance gene product, AarC) was detected which catalyzes conversion of succinyl-CoA to succinate (Table 2 and Figure 6) (Mullins et al., 2008, 2012; Azuma et al., 2009). This alternative bypass appears to preserve the typical cyclic course of TCA, allows acetate incorporation without substrate level phosphorylation and enables the removal of diffusively trapped cytoplasmic acetate by acetyl-COA oxidation which is considered as a detoxification step. As it is seen in Table 2, SCACT was significantly up-regulated while *Acetobacter senegalensis* LMG 23690^T^ was acclimated to EtOH condition or GlcAA condition.

It has been thought that a modified TCA cycle plays a key role in acetate oxidation making the cells resistant to acetic acid (Matsutani et al., 2011). In one study on *Acetobacter aceti* 1023, it was shown that AarC and AarA had the highest activity during ethanol oxidation and acetate overoxidation phase, which is consistent with a role for variant TCA cycle in dissimilatory acetate metabolisms (Mullins and Kappock, 2013). It is also believed that the levels of TCA cycle enzymes are correlated with acetic acid production and also resistant to acetic acid (Mullins and Kappock, 2013). As a result, the up-regulation of TCA enzymes under the conditions of the present study suggests that the production of biomass by a mixture of glucose and ethanol or a mixture of glucose and acetic acid as carbon sources, induced the acetic acid tolerance. Interestingly, even the levels of some TCA cycle enzymes such as SCACT, oxoglutarate dehydrogenase and isocitrate dehydrogenase were higher in GlcAA condition than in EtOH condition meaning that, cells acclimated to GlcAA condition, are probably more resistant to acetic acid.

In a previous study, we showed that using glucose at low pH (condition Glc) did not generate cells able to resume growth on ethanol and acetic acid (Shafiei et al., 2013a). The findings of the present study confirm those observations, because the levels of up-regulated enzymes which are presumably involved in acetic acid metabolism and detoxification were significantly lower in cells acclimated to glucose.

**FIGURE 7:**
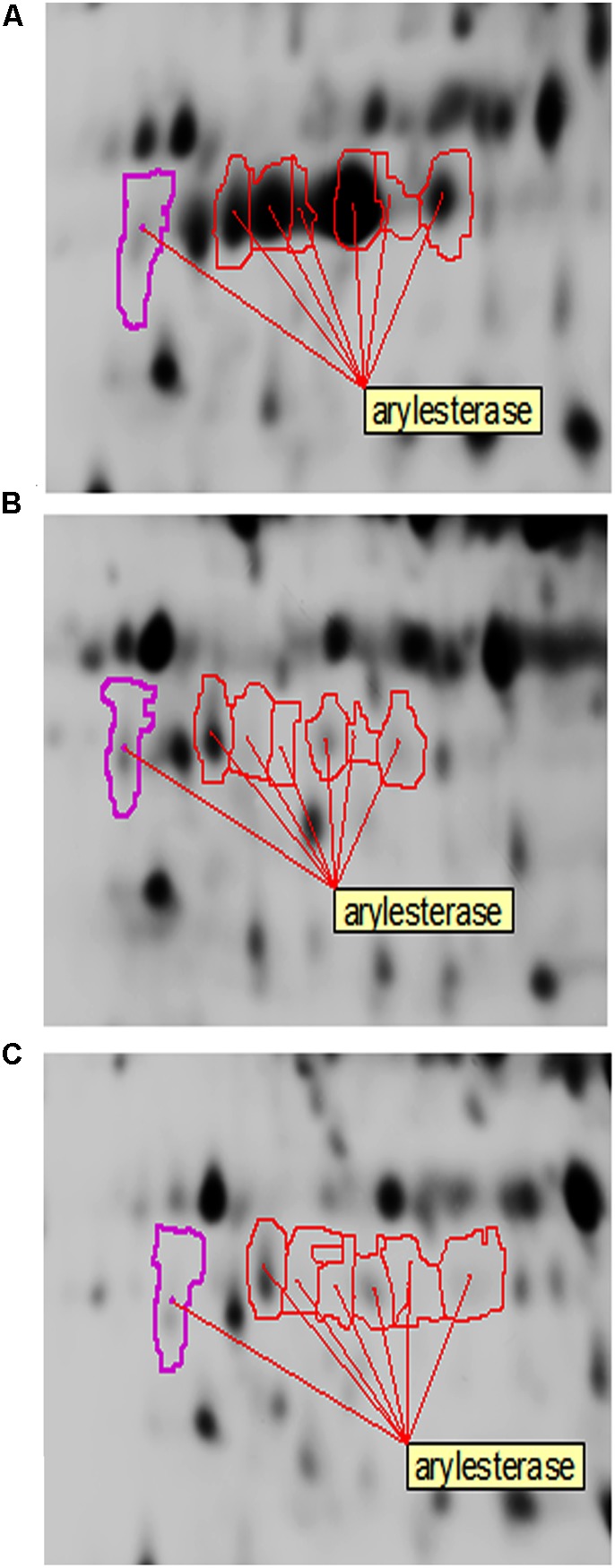
Changes of arylesterase isoforms of *A. senegalensis* LMG 23690^T^ acclimated to different carbon sources. **(A–C)** Show equivalent regions of 2-DiGE gel separations of CyDye-labeled proteins obtained from cells acclimated to ethanol, glucose and a mixture of glucose and acetic acid, respectively.

In the present study, arylesterase is one of the most highly up-regulated enzymes in the presence of ethanol (EtOH condition) with several identified isoforms of which five are strongly up-regulated in the presence of ethanol (Table 2 and Figure 7). However, as shown in Figure 7, they did not show significant up-regulation in the presence of glucose or acetic acid. Arylesterase catalyzing ethyl acetate formation (Figure 6), is induced by ethanol in *Acetobacter pasteurianus* (Sievers and Swings, 2005). In addition, it has been shown that esterification of ethanol and acetate is favored in low O_2_ concentration (Ory et al., 1998). In this study, expression of arylesterase, may reflect O_2_ deficiency during acetous fermentation. Moreover, because arylesterase contributes in aroma production during acetous fermentation (Sievers and Swings, 2005), it provides *Acetobacter senegalensis* LMG 23690^T^ with the capacity of softening the strong smell of acetic acid in vinegar.

### Proteins Involved in Oxidation–Reduction Process

In addition to the proteins involved in universal stress response, we observed that other proteins related to oxidation–reduction process such as osmotically inducible protein C (OmsC) (involved in the cellular defense mechanism against oxidative stress) (Park et al., 2008), superoxide dismutase (SOD) and thioredoxin (Trx) were overexpressed in the presence of acetic acid or ethanol (Table 2 and Supplementary Table S1). These observations are consistent with previous study on *Acetobacter pasteurianus* showing that the up-regulation of those proteins occurred in the presence of ethanol or endogenous acetic acid (Andrés-Barrao et al., 2012).

Superoxide dismutase was only slightly up-regulated in the presence of ethanol which is also an indication of oxidative stress, but the comparison between cells acclimated to glucose with cells acclimated to a mixture of glucose and acetic acid, showed a significant difference between those conditions. Sakurai and coworkers showed that SOD was highly over expressed in *Acetobacter aceti* NBRC 14818 growing exponentially in the presence of glucose while lower expression of SOD was observed in acetic acid and ethanol (Sakurai et al., 2011).

## Conclusion

In the present study, we investigated the influence of acclimation to different carbon sources on the ability of *A. senegalensis* LMG 23690^T^ cells to tolerate stresses related to downstream process. It was demonstrated that the combination of sequential acclimation with fermentation under sub-lethal stress condition enabled *A. senegalensis* LMG 23690^T^ to overcome some stress conditions such as freeze-drying process. Since cells acclimated to EtOH condition were non-viable after harvesting process, they were not suitable for freeze-drying process. However, acclimation to a mixture of acetic acid and glucose in low regulated pH (3.9) (condition GlcAA) caused a cross protection which enabled cells to grow in culture medium containing ethanol (EtOH condition), and resist against stress such as freeze-drying process. Results of proteome analysis by 2D-DiGE also showed similarities between the differentially expressed proteins of acclimated cells to EtOH condition and the proteome of acclimated cells to GlcAA condition. Practically, by acclimation to GlcAA condition, we achieved a qualified biomass which (i) showed an improved tolerance to freeze-drying process (ii) oxidized ethanol rapidly after rehydration. In order to produce a cost-effective starter, further studies are necessary to optimize the downstream process for preservation of acclimated cells.

## Data Availability

All data generated or analyzed during this study are included in this published article and its Supplementary Information Files.

## Author Contributions

RS designed the experimental setup; carried out the fermentation, downstream process, and proteomic analyses; and prepared the manuscript, figures, and tables. PL is a Senior Research Associate of F.R.S.-FNRS supervised the proteomic analysis; assisted with the preparation of figures and tables; and revised the manuscript. AS contributed in evaluating the phenotypic features of acclimated cells, and helped in the revision of the manuscript. PT and FD supervised the whole work and revised the manuscript. All authors read and approved the final manuscript.

## Conflict of Interest Statement

The authors declare that the research was conducted in the absence of any commercial or financial relationships that could be construed as a potential conflict of interest.
